# Provision of dementia-specific care in nursing homes in North Rhine-Westphalia (Germany) – analysis of person-centered practices and related problems within a holistic multiple case study

**DOI:** 10.1186/s12912-025-02726-5

**Published:** 2025-02-01

**Authors:** Kathrin Schmüdderich, Anne Fahsold, Jonas Dörner, Martina Roes, Rebecca Palm, Bernhard Holle

**Affiliations:** 1 German Center for Neurodegenerative Diseases (DZNE), Witten, Germany; 2 Faculty of Health, School of Nursing Science, Witten/Herdecke University, Witten, Germany; 3 School of Medicine and Health Sciences, Carl Von Ossietzky Universität Oldenburg, Oldenburg, Germany

**Keywords:** Dementia-specific care, Person-centered care, Delivery of healthcare, Advancing nursing care, Residential facilities, Qualitative research, Case studies, Needs assessment

## Abstract

**Background:**

To ensure high-quality care for residents living with dementia, recommendations for dementia-specific care do exist internationally as well as in Germany. Nevertheless, it remains unclear how dementia-specific care is currently provided and what can be derived from this for the improvement of dementia-specific care. Therefore, this study aimed to investigate the provision of dementia-specific care and related problems in German nursing homes.

**Methods:**

We used a holistic multiple case design with a total of four cases. The cases were defined as care units in which residents living with dementia were cared for. For data collection, we used problem-centered face-to-face interviews, document analysis, and context questionnaires and analyzed all qualitative data inductively and deductively using content structuring qualitative analysis. To identify case-specific and cross-case patterns and themes, we focused on similarities and differences between the cases. The reporting followed the EQUATOR reporting guideline for organizational case studies.

**Results:**

We interviewed 21 professionals, 14 relatives and 8 residents living with dementia. Despite context-specific differences, we identified a variation of care practices and problems in applying person-centered, dementia-specific care in German nursing homes. In all cases, these belong to the following topics: 1) *handling neuropsychiatric symptoms,* 2) *dealing with communication difficulties,* 3) *providing person-centered interaction and communication,* 4) *dealing with stress caused by experiencing dementia-specific symptoms* and 5) *using and sharing knowledge*. Even though the problems were identified in all cases, we also found differences in the extent and perception of these problems across the analyzed cases.

**Discussion:**

Despite existing conceptual recommendations and described care practices in our study, the identified problems showed that current care practices are perceived as problematic and partly are not person-centered. This highlights that person-centered requirements in dementia-specific care are not yet adequately addressed and that there is a need to give greater consideration to the identified problems when developing interventions to improve quality of care. Furthermore, the identified context-specific differences in the extent and perception of these problems show that the designs of new care models should allow for more flexibility, so that written recommendations can be implemented in practice and adapted to given contexts.

**Supplementary Information:**

The online version contains supplementary material available at 10.1186/s12912-025-02726-5.

## Background

The number of people living with dementia in German nursing homes is high and is expected to further increase due to demographic and social developments [1, 2]. As a result, nursing home care is becoming more and more complex. At the same time, the majority of care in German nursing homes is provided by nursing staff, about 50% of whom have a nursing-specific vocational training (three years of non-academic education to become a registered nurse) [3]. These nurses with at least three years of non-academic education will be called “nurses” in the whole article. The other half of the nursing staff employed in German nursing homes are nursing assistants, who have one to two years’ of non-academic nursing or non-specific training, or non-qualified staff. The proportion of "specialized nurses" in nursing homes (nurses with three years of non-academic education and one to two years of additional training, e.g., in geriatric-psychiatry, or academically trained nurses with a bachelor’s or master’s degree) is currently less than 1% of the nursing staff [3]. Due to recent political developments (e.g., a new mandatory tool for calculating nursing staff in German nursing homes) it is expected that the proportion of nurses and nursing specialists will continue to decrease in future, whereas the proportion of nursing assistants will continue to increase [4].

To be able to provide high-quality dementia-specific care for residents living with dementia, the general context conditions in German nursing homes, such as different qualification levels of nursing staff (nurses, specialized nurses, nursing assistants), need to be viewed, analyzed and evaluated within other specific requirements for dementia care. Therefore, it is necessary to consider a new skill mix and new role allocations that are in line with the complexity of dementia care. This offers the opportunity to create prospects for staff development and to ensure high-quality intra- and interprofessional care for residents living with dementia with complex care needs by advancing nursing practices [5, 6]. One way to achieve these goals may be to develop and implement a nurse-led care model. Nurse-led care models in residential care are characterized by the fact that residents' care, from assessment to evaluation, is steered and coordinated independent and within the responsibility of a nurse with an advanced skill set. Here, coordination of care refers to the collaborative work of several actors [7]. To develop a tailored nurse-led and dementia-specific care model for German nursing homes, this model has to take into account both organizational and contextual challenges as well as content-related requirements of dementia care.

With respect to the requirements of dementia care, there are existing recommendations in regulations and guidelines that describe high-quality dementia care. Internationally, these include for example the Dementia Care Practice Recommendations by the Alzheimer’s Association for quality care practices that illustrate that the person-centered focus is the core of quality dementia care [8]. For Germany, regulations and guidelines for dementia care include the S3 guideline on dementia, which focuses primarily on medical diagnostic procedures and treatments [9], as well as the National Expert Standard "Fostering and sustaining relationships in care for people living with dementia”, which focuses primarily on nursing and caring [10]. National Expert Standards can be used to define, implement and evaluate the quality of performance. Therefore, they highlight the specific contribution of nursing care to the health care of patients, residents and their relatives with regard to key quality risks and offer a basis for continuous improvement in the quality of care in healthcare facilities and nursing homes [11]. The National Expert Standard mentioned above embeds relational specific care within person-centered dementia care and describes criteria to evaluate the care of people living with dementia according to the person-centered care approach by Kitwood [12]. This approach gives priority to the person and highlights possibilities for high-quality interactions to understand the person living with dementia and their psychosocial needs [10, 12, 13]. Comparable to international recommendations [8], the criteria described in the National Expert Standard include, for instance, training courses, psychosocial intervention approaches and aspects of the environment and milieu design that are intended to maintain or promote the feeling of people living with dementia to be heard, understood and accepted as well as to be connected with other people [10].

The idea behind the National Expert Standard was to achieve significant quality improvements in dementia care [10]. However, the implementation of the National Expert Standard is not obligatory, and sustainability in practice has thus far been rated well, to varying degrees [14]. Further, there have been reviews internationally to reflect on the implementation of person-centered intervention aspects in nursing homes, concluding that a successful implementation of person-centered care remains challenging and is associated with different prerequisites and contextual conditions [15, 16]. Nevertheless, studies on the implementation of person-centered intervention aspects in German nursing homes are limited: a mini intervention to use information about meaningful situations in planning and providing care [17], dementia care mapping [18], dementia-specific case conferences [19] and experts in person-centered care for the elderly [20]. Next to that, few international studies have investigated how dementia-specific care is currently provided and organized in nursing homes and which problems are associated with that [21, 22]. Although few studies in Germany also identified organizational characteristics of usual and dementia-specific care units [23–25], there are no studies to date that describe in detail the current provision of dementia-specific care and associated problems in Germany.

Therefore, what remains unclear is how dementia-specific care for people living with dementia is provided in different nursing home contexts and which problems persist in the care of residents living with dementia in German nursing homes. Nevertheless, this understanding of daily practices and problems as well as context-specific differences is relevant for developing a tailored dementia-specific, nurse-led care model that can be implemented under real-life conditions and improve dementia care [7, 26]. Furthermore, this knowledge can help to reveal existing problems in dementia care even more explicitly – also for the international research landscape – to be able to discuss appropriate solutions for providing high-quality dementia care.

Therefore, the aim of this study was to explore how dementia-specific care is provided in German nursing homes and to investigate the problems associated with current dementia-specific care in different nursing home contexts to derive relevant topics for the development of a dementia-specific, nurse-led care model.

Our research questions were as follows:

- *How are dementia-specific requirements currently dealt with in the care of residents living with dementia in different nursing homes in Germany?*
- *What aspects are perceived as problematic regarding current care delivery in different nursing homes in Germany from the perspectives of residents living with dementia, relatives and professional staff?*

## Methods

The reporting of this case study followed the EQUATOR (Enhancing the QUAlity and Transparency Of health Research) reporting guideline for organizational case studies [27] (s. Additional File 1). No study protocol was registered.

### Design

This study is part of a qualitative multiple case study that also examined professional collaboration in the care of people living with dementia [6]. The design of the case study corresponds to a holistic multiple case design according to Yin [28]. This design is characterized by the consideration of multiple cases (we included four cases), which are all analyzed holistically. The design thus enables a comprehensive, in-depth examination of the individual cases (holistic analysis) as well as the examination of similarities and differences between the cases (multiple cases) [28].

The cases were defined organizationally at the care unit level so that each case represented a real-world organization [28, 29]. The period of data collection (February to August 2022) and the context of the nursing homes as well as their affiliation with the federal state of North Rhine-Westphalia (Germany) formed the temporal and spatial boundaries of the cases [6].

A holistic multiple case study was suitable for this research objective, as including multiple cases helped to get more robust findings and made it possible to explore and compare various care realities during the data analysis. Dealing with dementia-specific requirements was further analyzed holistically, taking into account different perspectives and contexts for each case to achieve a holistic understanding of current care practices and existing problems [28]. Further, the holistic design was chosen to stay focused on our research interest to examine the provision of dementia-specific care and related problems in different nursing home contexts.

### Study setting and participants

Representatives of organizations, including nursing homes the DZNE (Deutsche Zentrum für Neurodegenerative Erkrankungen) Witten collaborates with, received information about the study and the conditions for participation via email. The nursing homes that were interested in participating contacted the researchers and got further details about the study. According to Yin [28] and Creswell [30], we purposefully selected four care units from different nursing homes located in the federal state of North Rhine-Westphalia that represented typical care units in German nursing homes (typical cases) and served as replication of the research question and method. When selecting the care units, we tried to achieve a certain heterogeneity of the care units (variations in the size, ownership and location of the care units). However, the association to the federal state of North Rhine-Westphalia was the only fixed inclusion criteria. We further included both usual care units and dementia-specific care units. Other specific types of care units (e.g., palliative care units, green farms) were excluded. Potential participants were identified and informed by the nursing home or care unit manager using a recruitment form as well as written information. If potential participants were interested in participating, the researchers were contacted by the nursing home manager.

Our participants were residents living with dementia, relatives of residents living with dementia and internal and external professionals. To ensure that we included the perspectives of the residents living with dementia, our inclusion criteria for residents was, that they had to have both a medical approved dementia diagnosis and cognitive impairment according to the Dementia Screening Scale (DSS) [31]. This means that the residents needed to have a DSS score above two [32]. Moreover, only residents who had lived in the selected care unit for at least four weeks prior to data collection were included. Regarding relatives, we only included relatives of residents living with dementia of the selected care unit. Further, we only included professionals, who 1) were working in (internal professionals) or with (external professionals) the selected care unit, 2) were providing care for residents living with dementia of the care unit, 3) had at least one year of professional experience, and 4) had an educational training in a care-related field. This included nurses with a minimum of three years of education, social workers, physicians, therapists as well as pharmacists. All professionals with less than three years of educational training in a care-related field were excluded from the study. Further, all interview participants with insufficient German language skills or the inability to interact in an interview situation were excluded. For residents living with dementia, this meant that communication difficulties per se were not a reason for exclusion, although the ability to interact with the researcher in an interview situation with the support of their relatives was a requirement. In those cases where an interview with the resident living with dementia was not possible, we included the resident's file in our document analysis.

### Data collection

The data collection process took place from February to August 2022, and all the data were collected either in a quiet room in the nursing home or in the physicians’ practice. We used different data sources to describe and explore the cases [28, 30].

First, we conducted face-to-face problem-centered interviews [33, 34] with residents living with dementia and relatives of residents living with dementia as well as problem-centered expert interviews [35] with internal and external professionals. Using Helfferich's method of collecting, checking, sorting and subsuming questions [36], interview guidelines were developed for each interview type (residents living with dementia, relatives and professionals) on the basis of the National Expert Standard “Fostering and sustaining relationships in care for people living with dementia” [10] to ensure that all relevant topics were discussed (s. Additional File 2). The interviews were supplemented by clarification questions, a postscript and short questionnaires to collect data on sociodemographic characteristics, the health-specific characteristics of the residents and the job-specific characteristics of the professionals [33, 34]. We conducted one pretest for each interview guideline. All interviews were then digitally audio-recorded and transcribed by an external transcription service. To make the interview situation as stress-free as possible for residents living with dementia, these interviews were conducted together with a relative or a trusted person, which could also have been a staff member. To prevent the relatives from emphasizing their perspective during the interviews with residents living with dementia, they were interviewed immediately before the interviews with the residents. All other interviews were designed as individual interviews to encourage the participants to talk openly about their experiences and wishes.

As a second data source, the first author analyzed the nursing records of the residents living with dementia and documented selective passages of the care plans and daily reports from the last six months as verbatim quotes that focused on dementia-specific aspects of care. For this purpose, a template that covered the topics of the National Expert Standard was used. This included the same categories as the interview guidelines (s. Additional File 2): involvement of residents living with dementia and relatives; interaction, communication and relationship building; problems in dementia-specific care; and care activities and measures for dementia-specific care. Since the documents could only be made available for a short period of time, this procedure corresponded to an extraction of the data in relation to predefined topics for subsequent coding [37]. The document analyses were carried out with those residents living with dementia with whom or with whose relatives the interviews were conducted. Following Yin [28], this served to understand, corroborate, and augment the evidence from the interviews and context questionnaires.

Third, we used context questionnaires for the case descriptions that included structural, financial, staff- and resident-specific aspects of the care units and nursing homes (s. Additional File 3) and the Dementia Care Questionnaire (DemCare-Q) [38] to collect data on dementia-specific interventions that are already implemented at the resident level. The context questionnaire and the DemCare-Q were assessed in conversations with the nursing home manager and sociodemographic questions for the sample description were answered by the participating relatives or professionals before the interviews started. Questions about the care of the residents living with dementia as well as sociodemographic and health-specific aspects were answered during conversations between the researcher and the participating residents or the nurses, who were responsible for the resident’s care and by referencing the resident’s file.

### Data analysis

Following Yin [28], all the data were first merged at the case level for the data analysis. The quantitative data were analyzed descriptively for sample description. All other standardized data were analyzed narratively to provide case context descriptions and to supplement the qualitative data.

The qualitative data were then analyzed following the individual steps of the content-structuring analysis by Kuckartz and Rädiker [39]. Using an inductive-deductive approach, the first author developed main categories based on the interview guideline. These main categories were initially independently coded by KS and JD in three interviews. Afterwards, they were discussed, revised and complemented with inductive main categories as well as specified in category definitions and tested in another two interviews. Once a common understanding of the main categories was developed, KS coded all the interviews and developed subcategories inductively for each case to identify case-specific themes and patterns (within-case analysis). Passages of the nursing records (care plans or daily reports) were either coded to existing categories or shaped new categories, and descriptive material from the context questionnaires (context units) was consulted to better understand single coding units. To discuss and clarify subcategories, category definitions and subjective impressions, JD (*Case C*) and AF (*Case A*) also coded one case each. This coding was done independently, but with intermediate discussions. On the basis of the qualitative categories and the standardized data, we created tabular overviews and case-specific thematic summaries for each case (case descriptions) [28, 39].

Subsequent to the within-case analysis, we performed a comparative analysis of the cases (cross-case analysis) using the developed tabular overviews that were based on the qualitative categories and case descriptions to identify both similarities (cross-case characteristics and themes), as well as differences between the individual cases [28]. Finally, we created visualizations of the interrelationships of the categories as well as cross-case differences to illustrate our findings.

We analyzed all qualitative data with MAXQDA 2022 [40] and all quantitative data with SPSS Statistics V21 [41].

### Ethical considerations

Participation in this study was voluntary, and informed consent was obtained from all participants or their legal representatives prior to data collection. Additionally, ongoing consent was obtained from the residents living with dementia before and during the interviews. Ethical approval was obtained from the German Society for Nursing Science (DGP e. V.) (registration number 21–032).

### Us of artificial intelligence (AI)

To present original data in this paper, the first author used DeepL (https://www.deepl.com/translator) free version to translate interview quotations from German to English. The translation of the quotations claimed to reproduce the content of what was said accurately. After using this tool, the first author reviewed and discussed the translations with the other authors and edited the content as needed. The first author takes full responsibility for the content of the publication. We did not use any other AI tools.

### Rigor

All the authors involved in the data collection and analysis are research associates with a background in nursing and experience in the care of residents living with dementia. We addressed *credibility* by 1) conducting the coding process in pairs (KS and JD; KS and AF) and 2) discussing the study design, the results and their presentation with senior researchers (MR, RP, and BH), with an interdisciplinary methods group for qualitative research as well as with international peer groups of PhD students and experienced supervisors (*reliability*). *Confirmability* and *dependability* were achieved through 3) triangulations between researchers and data sources, 4) a logbook to reflect on our preconceived notions and decisions throughout the study, and 5) the use of citations from the original data (*reliability, construct validity*). Furthermore, *transferability* was obtained through 6) a transparent and comprehensive reporting to allow readers to interpret and evaluate the results for their own contexts (*external validity*) [28, 42]. We did not assess aspects of *internal validity* as recommended by Yin [28], as this was not an explanatory study.

## Results

**Fig. 1:**
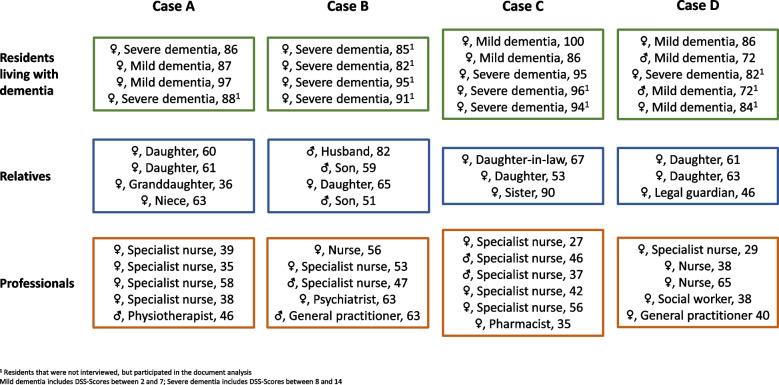
Characteristics of the participants included in each case

In total, we included four care units from four different nursing homes in North Rhine-Westphalia. Our data comprised four context questionnaires, 18 resident files from residents living with dementia and 44 interviews with different stakeholders: 21 professionals (nurses, physicians, social worker, physiotherapist, and pharmacist), 8 residents living with dementia and 14 relatives of residents living with dementia (Fig. 1). The length of the interviews was 31—128 min (mean 69 min) for professionals, 10—89 min for relatives (mean 47 min) and 10—26 min (mean 16 min) for residents living with dementia.

### Case description

All of the included nursing homes are located in a semiurban to urban region and have two to three structurally separated care units. None of the care units is a care unit with secured exit or access controls. Case conferences and pain assessments are carried out in all care units, whereas behavior, dementia severity or the presence of depression are not assessed in any of the care units. In addition to these similarities, few structural and organizational aspects differ across the cases (Table 1). The characteristics of the cases are illustrated in the following case descriptions as part of the results of the within-case analysis.

**Table 1:** Characteristics of the nursing homes and care units

	**Case A**	**Case B**	**Case C**	**Case D**
**Nursing Home**				
Number of beds	87	32	63	100
Dementia-specific care concept	Yes	Yes	-	Yes
Person-centered care (T. Kitwood)	Yes	Yes	-	-
Validation (N. Feil; N. Richard)	-	Yes	-	Yes
**Care Unit**				
Care focus agreed with cost unit	-	Dementia	-	-
Staff				
RNs (3-year nursing education), FTE (n)	8.00 (10)	2.04 (4)	6.00 (7)	2.75 (3)
RNs with further training, FTE (n)	3.00 (3)	3.65 (6)	1.00 (1)	2.00 (2)
Nursing assistants, FTE (n)	-	2.05 (4)	1.35 (2)	1.00 (1)
Unskilled nurses, FTE (n)	10.00 (14)	3.55 (8)	4.35 (6)	5.25 (6)
Nursing trainees, FTE (n)	2.00 (2)	2.00 (2)	-	3.00 (3)
Residents				
Residents in the care unit, n	47	22	26	28
Care level 2, in %	14.90%	-	-	17.86%
Care level 3, in %	42.55%	9.09%	50.00%	57.14%
Care level 4, in %	31.91%	72.73%	23.08%	17.86%
Care level 5, in %	10.64%	18.18%	26.92%	7.14%
Dementia diagnoses, in %	42.55%	100.00%	26.92%	39.29%
Judicial accommodation or physical restraint measures	-	63.64%	34.62%	10.71%
Care and support				
Case conferences	Yes	Yes	Yes	Yes
Dementia Care Mapping	-	Yes	Yes	-
Pain assessment	Yes, for all	Yes, for most	Yes, for all	Yes, for all
Behavior assessment	-	-	-	-
Dementia severity assessment	-	-	-	-
Quality of life assessment	Yes, for all	Yes, for all	-	-
Depression assessment	-	-	-	-

*RN* Registered Nurse, *FTE* full-time equivalent (indicates a full-time position in the care unit)

***Case A*** refers to a care unit that comprises 47 residents. Approximately 43% of the residents have a medical approved diagnosis of dementia. None of the residents receives judicial accommodations or physical restraint measures. Thirteen nurses (full-time equivalent = 11.00) care for the residents of the care unit. Three of these nurses have a specialization in palliative care, mentoring, nursing management, or geriatric-psychiatry. A written dementia-specific care concept exists for the nursing home, which is based on the person-centered approach of Kitwood. This concept includes case conferences, validation, biography work, multisensory stimulation, promotion of mobility, milieu therapy and counseling as measures. Care activities that are carried out in the care unit include for example table bowling, cocktail and wellness afternoons as well as singing groups and pony visits. Biographies are queried by the nursing home manager as part of an integration interview when moving in.

***Case B*** refers to a dementia-specific care unit that has negotiated funding of higher costs for more and dementia qualified nurses according to the Social Care Law (§§ 84, 85 SGB XI – Social Code Book XI). This unit includes 22 residents. 100% of the residents living in this care unit have a medical approved diagnosis of dementia, and approximately 64% receive judicial accommodations (e.g., GPS wristband) or physical restraint measures (e.g., bed restraint). Ten nurses (full-time equivalent = 5.69) care for the residents living with dementia of the care unit. Six of these nurses have a specialization in palliative care, mentoring, or geriatric-psychiatry. A written dementia-specific care concept exists for the nursing home, which is based on the person-centered approach of Kitwood and the validation approach according to Naomi Feil. This concept includes ritualized communication, case discussions, validation, biography work, multisensory stimulation, promotion of mobility, and kinesthetic as measures. Furthermore, Dementia Care Mapping (DCM) observations take place regularly. Care activities that are carried out in the care unit include for example ball games, walks, baking activities and seasonal themed events. Biographies are queried by the nursing home manager as part of an integration interview when moving in.

***Case C*** refers to a care unit that has negotiated additional funding for a provider specific house community concept according to the Social Care Law (§§ 84, 85 SGB XI), which mentions the inclusion of more housekeeping staff and working in small care groups for a resident-oriented day structure. Approximately 27% of the residents living in this care unit have a medical approved diagnosis of dementia, and approximately 35% of the residents receive judicial accommodations or physical restraint measures. Eight nurses (full-time equivalent = 7.00) care for the residents of the care unit. One of these nurses is a wound expert. A specialized nurse in geriatric-psychiatry, who works not on the included care unit, consults the whole nursing home. The nursing home does not have a written dementia-specific care concept, which applies for the nursing home. Nevertheless, an external professional performs DCM observations regularly. Care activities that are carried out in the care unit include for example handicrafts, gymnastics, game afternoons and religious activities. Biographies are queried by the social worker as part of an information exchange when moving in.

***Case D*** refers to a care unit that includes 28 residents. Approximately 40% of the residents living in this care unit have a medical approved diagnosis of dementia, and approximately 11% of the residents receive judicial accommodations or physical restraint measures. Five nurses (full-time equivalent = 4.75) care for the residents of the care unit. Two of these nurses have a specialization in palliative care and mentoring. The nursing home does not have a specialized nurse in geriatric-psychiatry. A written dementia-specific care concept exists for the nursing home, which is based on the validation approach according to Nicole Richard. This concept includes case discussions, validation and biography work as measures. Care activities that are carried out in the care unit include for example bowling, memory groups, puzzle rounds and bingo. Biographies are queried by the social worker as part of an information exchange when moving in.

### Care practices and problems in dealing with dementia-specific requirements

Although we found many care practices to provide person-centered dementia care in our qualitative analysis, few ongoing problems were also identified in all cases. The existing care practices and problems relate to the following topics: 1) *handling neuropsychiatric symptoms*, 2) *dealing with communication difficulties*, 3) *providing person-centered interaction and communication*, 4) *dealing with stress caused by experiencing dementia-specific symptoms,* as well as 5) *using and sharing knowledge*. When analyzing the problems identified in our study through the lens of person-centeredness as represented in the National Expert Standard, we found that fostering and sustaining relationships with residents living with dementia is perceived as one of the key issues. This appears to be primarily due to the progression of dementia symptoms and feelings of being helpless to provide tailored, individual care according to the residents’ needs.

**Fig. 2:**
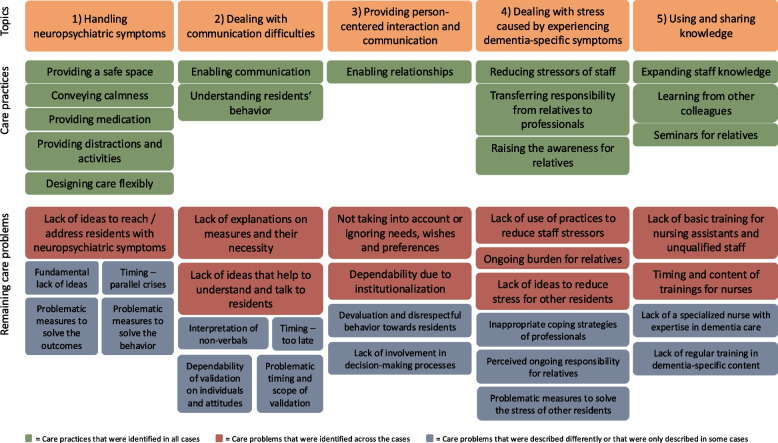
Identified care practices and related problems

Below, we illustrate our results according to the identified topics (Fig. 2). For each topic, we firstly describe existing care practices that were identified to deal with dementia-specific requirements and to provide person-centered dementia care. These care practices were described as well-known and used in everyday care. Secondly, we outline the associated problems experienced by the interviewees or documented in the records by the professionals. All these results are part of the cross-case analysis, illustrating similarities as well as differences between the cases.

#### 1) Handling neuropsychiatric symptoms

Cognitive impairments and neuropsychiatric symptoms associated with dementia are perceived as challenging by relatives and professionals *in all cases*, as they lead to changes in personality and impulse control as well as in the perception of reality and contribute to residents’ increased need for protection. *In all cases*, similar known and used care practices to address these requirements and to provide person-centered dementia care were described by relatives and professionals. These include:

- Providing a safe space (ensuring care; minimizing danger; creating a familiar environment; providing (daily) structure and continuity)
- Conveying calmness (taking time; radiating calm and actively listening; reducing stimuli)
- Providing medication (giving medication as needed; referring to psychiatry)
- Providing distractions and activities (offering care activities; distracting with social activities)
- Designing care flexibly (waiting for phases and trying later; changing the person or procedure; individualizing offers)

Despite these existing care practices, problems in handling neuropsychiatric symptoms were identified by professionals and relatives *in all cases*. What works or does not work feels often like random luck for the professionals, and there are few ideas on what to offer, how to distract someone or how to respond to feelings and emotions in critical situations. *In all cases*, this gives professionals a feeling of insecurity and helplessness. "*When I ‘ve applied all my ideas and they don’t work, then I'm left looking stupid*" (Case A, professional 2). Daily mood swings and individual personalities of residents living with dementia further complicate general measures and forward-looking planning. In particular, agitated and aggressive behaviors, walking tendencies and defensive behaviors were described as difficult to handle *across all cases*. A failure to address agitation, aggression and walking tendencies significantly increases the self-endangerment of residents living with dementia as well as the risk of harm to others. Further, unaddressed defensive behavior can contribute to the inability to obtain necessary medical tests or care and to the need to administer medication on suspicion, which may worsen the general conditions of the resident (*Cases B, C*). On the other hand, restraining residents with walking tendencies or carrying out care despite residents displaying defensive behavior often results in increased agitation and aggression. Overall, these problems in handling neuropsychiatric symptoms lead to increase feelings of failure and helplessness *in all cases*, since it seems either that the staff cannot reach the residents or applied psychosocial interventions are not successful for a particular resident.

*"On Sunday, I actually had a resident who was in a state of agitation […] I really thought I would have to call an ambulance […] nothing helped, validating conversations, calming conversations, staying with him… Until he was exhausted and three of us could put him to bed"* (Case C, professional 14).

##### Differences between the cases

Comparing the cases, in *Cases A, C* and *D*, there seems to be – next to inadequate contextual conditions (such as lack of staff and time) – a fundamental lack of measures and ideas for dealing with the broad variation of neuropsychiatric symptoms. This leads to staff being overwhelmed and – depending on individual staff – inadequate reactions to neuropsychiatric symptoms, such as arguing with residents living with dementia, ignoring their behaviors or quickly administering medication without any reflection (*Cases A, C*).

*„If someone faces me very agitated, then I could or I should actually take the time to find out what is going on with him at the moment. Then, I would do him more justice. I don’t always do that. Actually, I can only ever try this to a certain extent “* (Case C, professional 15).

In contrast to *Cases A, C* and *D*, the problems in *Case B* are primarily based on the fact that residents’ crises cannot always be addressed at an early stage, which means, that conventional well-known care practices cannot be applied anymore (*Case B*). The reasons for that are simultaneous crises involving several residents as well as missing strategies how to handle these situations lead to a feeling of lack of time.

„*So, most of the time you do even notice the problem, but your hands are really full with other work and the other work is another resident, usually. And it’s always very difficult, nearly impossible, to manage two residents in crisis individually* (Case B, professional 10).

Another difference across the cases is based on the overall understanding of these problems. While in *Cases A* and* D,* it is assumed that walking tendencies are problematic to handle but not avoidable, and a standard procedure has been established (such as calling the police and relatives to search for the resident), *Cases B* and* C* focus more strongly on trying to prevent residents from leaving the nursing home. They describe these attempts as well as the negative consequences as significantly more problematic. Similarly, noisy behaviors are experienced as less problematic for those affected in *Cases A* and* B*, so that the challenges in dealing with them relate primarily to raising awareness of relatives and roommates in the context of crisis talks. However, in the other cases, the inability to reduce the noisy behavior is seen as problematic for both the affected person and their environment. In these cases, measures to exclude these residents living with dementia from the group were described as helpful and positive for themselves and others (*Cases C, D*).

#### 2) Dealing with communication difficulties

Due to communication difficulties, conversations with residents living with dementia and the identification of their needs are perceived as difficult by relatives and professionals *in all cases*. Therefore, a different type of communication becomes necessary. Known care practices described and used by relatives and professionals to address this are similar *in all cases* and include:

- Enabling communication (choosing appropriate, simple words; addressing residents by their first name and with the informal personal pronoun, which is normally used for people you are familiar with on a personal level (family, friends); using biographical aspects; talking about everyday life; communicating nonverbally; validation and, in *Case B*, ritualized communication, a form of integrative validation, which is based on ritualized sentences or touches at the beginning and end of each interaction),
- Trying to understand residents’ behavior (trial and error; changing the perspective)

Nonetheless, *in all cases* both professionals and relatives describe problems in understanding residents’ needs. These problems are prevalent when residents can no longer express themselves verbally and their gestures and facial expressions cannot be interpreted (*Cases B, C, D*) or when residents are already in an acute crisis and are difficult to reach (*Cases A, B*). Outcomes that are perceived problematic include having to accept that the need will not be understood or continuing to experiment without knowing what the specific problem is (*Case C*). For residents living with dementia, this can lead to a feeling of being misunderstood, a loss of trust, anxiety, inner restlessness or aggression (*Cases A, C, D*).

*“In the afternoon, we noticed that Ms. H. had difficulties finding the right words and could not express herself clearly. She pointed to objects or called them something else if she did not know the words. She appeared somewhat distressed and hit her forehead with her hand"* (Case A, resident file 1).

Moreover, problems in communication are described *in all cases* when explanations of measures and their necessity fail to materialize. This leads to a measure being implemented even though the resident does not fully understand it and may feel pressured by it (*Cases A, B*). Defensive behaviors or aggressions can arise as consequences.

##### Differences between the cases

Finally, validation – as the best-known measure – is perceived as difficult to apply *in all cases*, even if validation is part of the written dementia-specific concept in *Case A* (only described as a measure), *Case B* (approach by Naomi Feil) and *Case D* (approach by Nicole Richard). Comparing the cases, *Cases A, C* and *D* criticize that the success of the measure depends on the attitudes of residents and staff. Accordingly, how residents react to validation and whether validation can be harmful or have the opposite effect to that intended is perceived as very individual. Similarly, an extensive knowledge base in validation techniques is seen neither as a prerequisite nor as a guarantee for successful implementation. Conversely, much information about the resident's biography and the formulations of validation are deemed necessary, which makes a targeted application impossible not only for nursing assistants and non-qualified staff (such as supporting every-day activities and cleaning) but also for nurses in acute situations. The perceived lack of meaningfulness of this measure ultimately contributes to the fact that validation is not consistently practiced as a measure in *Cases A, C* and *D* or that the attempted implementation is even ridiculed (*Case D*).

"*Of course, we have examples for every situation. However, when the situation arises, I don't sit down and read through it first. Instead, I act intuitively. So, and some people here can do it better, others worse*" (Case A, professional 2).

Compared to the *Cases A, C* and *D*, *Case B* is less critical of the usefulness of the measure, but rather of the possible scope and timing of validation due to time and personnel shortages.

"*In validation you have to verbalize continuously […] [You should] not wait with offering validation until the crisis is already there. Instead, the validation offers should always maintain a wave of well-being, ideally from the beginning to the end of the shift*" (Case B, professional 10).

Besides that, *Case B* is the only care unit that uses ritualized communication. Therefore, ritualized sentences or touches to start and end an individualized conversation with each resident are defined and documented. Examples from the nursing record are the following: ritualized starting sentence such as “*good morning [name often the resident], the sportive lady from [city where she lived]*”; validating sentence “*you did a lot of sport in your life*”; ritualized closing sentence “*I also have to get back to work now, see you later*” (Case B, resident file 6). However, all nurses in *Case B* describe that this newer approach has not yet being well integrated into everyday care, which is why it is often forgotten.

#### 3) Providing person-centered interaction and communication

Due to cognitive and communicative limitations as well as neuropsychiatric symptoms and individual and daily changes in the conditions and moods of residents living with dementia, all participants perceive the ability of residents living with dementia to form relationships and advocate for themselves as limited. A common strategy known and used to address these requirements and to provide person-centered dementia care was mentioned *in all cases*:

 – Enabling relationships (encouraging participation; enabling self-determination; involving families; recognizing the person and being there for them)

Nevertheless, problems due to a lack of person-centered interaction and communication are pointed out by all participants *in all cases*. These include not taking into account or ignoring residents and their needs, wishes and preferences. The reasons cited include a lack of staff and time resources (*Cases A, C, D*), large groups of residents (*Case C*) and asymmetries. The latter contribute to 1) professionals or relatives acting against to the wishes of residents, as they think they know better (*Cases A, C*), or 2) dilemmas arising between staff-centeredness and resident-centeredness (*Cases B, C, D*). Furthermore, in *Cases A, C* and* D*, staff inattention and ignorance are described as factors that contribute to needs that are expressed verbally or nonverbally being disregarded or passed over.

*„Sometimes the resident doesn’t want [to participate]. […] he has been asked again and again and he said ‘no’ all the time. And at some point he left, because he just didn’t want to be part of it anymore […] After the fifth 'no', it was enough for him, then you shouldn’t bring him into the group. Yes, he left [the nursing home] via the stairwell “* (Case C, professional 14).

Throughout institutionalization, the residents are further subject to these situations and the underlying structures *in all cases*. This can lead to two extremes. Either, the residents and the relatives experience a lack of perceived adequate care and consideration of their individual needs and wishes: *„Once there was a man in my room. […] He washed me, went into the bathroom with me, just washed me and I didn't want that. […] I said so, and he didn't react at all “* (Case D, resident 7). This contributes to the residents not feeling comfortable and everyone involved being dissatisfied with the overall care situation: "*I’m at their mercy here, right? I’m dumped here. […] I don't have a home after all*" (Case A, resident 1). Alternatively, the expectations of those affected are strongly adjusted to the quality of care that is considered possible in a resignation: *„I only have what I need. I sit down there and then that's it. It’s boring […] I don’t have anything else. I don't chat much. What else can you do? “* (Case A, resident 2).

##### Differences between the cases

Comparing the cases, additional problems were identified in *Cases A, C* and *D* with respect to the attitudes of the staff. These are expressed in the criticism of devaluation and disrespect as well as in the lack of explanations of decisions for residents living with dementia. The devaluation and disrespect is demonstrated, for instance, in a disrespectful tone and insults when communicating with residents living with dementia (*Cases A, C, D*) as well as in the reduction of residents to their behavior (*Cases C, D*) and is described as a reaction to specific behaviors or the underlying dementia diagnosis (*Cases A, C, D*). The reasons for devaluation and disrespect stem not only from supposed ignorance but also from the specific personality traits of individual employees (*Case D*). As a result, this contributes to increased asymmetry and a lack of awareness of the person as an individual.

*„Decisions are simply made over their heads, right? Maybe the staff don’t mean it in a bad way. I mean, [for example the perceived temperature in the care unit: the staff] is on the move all day, which leads to them being more likely to be warm than an old person who just sits around all day […] and is therefore more likely to get cold. So when my mum says I want to wear a cardigan in the morning, the staff laugh and say you don't need it, it's warm outside"* (Case D, relative 12).

With respect to the self-determination of residents living with dementia, they are rarely or never involved in decision-making processes. Building on this, especially *Cases C* and* D* complain that decisions are not sufficiently explained to residents living with dementia, even retrospectively. The reasons described are that residents living with dementia are not expected to communicate this information themselves or it is assumed that they do not understand it (*Cases C, D*). As a result, this makes it difficult for residents living with dementia to understand changes in their care and reinforces their feelings of being ignored and not being taken seriously as a person.

#### 4) Dealing with stress caused by experiencing dementia-specific symptoms

Due to the diverse symptoms of dementia diseases, various care practices are used to relief both staff and relatives of people living with dementia. *In all cases*, the described existing care practices by professionals and relatives include:

- Reducing stressors of staff (having breaks and staff changes; debriefing; providing care with two professionals)
- Transferring responsibility from relatives to professionals (reducing feelings of guilt; taking responsibility for care coordination; creating relief and distance for relatives)
- Raising the awareness for relatives (recognizing and addressing emotions; trying to understand the relatives’ situation; protecting relatives from unpleasant situations)

Nevertheless, *in all cases*, a wide range of stresses caused by the experience of dementia-specific symptoms continues to be unaddressed, according to the participating professionals, relatives and residents. These affect staff and relatives as well as other residents of the care unit.

*In all cases*, problems in dealing with staff stress relate to the fact that either stress is not communicated within the team (*Case A*) or staff do not receive any breaks and are permanently exposed to intense stress factors (such as loud noises, aggressive or defensive behavior of the resident) (*Cases B, C, D*). This leads to anxiety, excessive demands, frustration, resignation, inadequate care and, above all, a high level of burden and dissatisfaction. The latter is reinforced, if the staff's own demands for high-quality care cannot be met due to a lack of measures, training or time to deal with dementia-specific symptoms.

Dealing with relatives stress is also identified as problematic *in all cases*. Despite the existing care practices of raising awareness for relatives, relatives feel rejected and hurt by the behaviors of the residents living with dementia or the fact that they can no longer remember shared experiences. Similarly, they have difficulties to communicate with their relatives, to connect with them and to accept changes in their life circumstances, personalities and roles within the family, even if these changes are generally understood (*Cases A, B, D*).

Finally, dealing with stress experienced by other residents of the care unit is described as problematic *in all cases*. Accordingly, witnessing dementia symptoms contributes to conflicts between residents and an aversion to people living with severe dementia.

##### Differences between the cases

When comparing the cases, the general conditions in *Cases C* and *D* (such as less staff, knowledge, and care practices), in particular, reinforce the fact that staff stress caused by experiencing dementia-specific symptoms cannot be addressed within the team. This further leads to the use of inappropriate coping strategies, which ultimately jeopardize the quality of care.

“*Individual support is often provided. With him, this is rarely the case. Many actually avoid him because he can quickly become aggressive. And mostly, care for him is carried out in pairs. Because one person has to hold him down so that the other doesn't get hit. And that’s really difficult, especially in terms of care, you try everything, but most of [the staff] avoid it* “ (Case C, professional 11).

Depending on the perception of support in the different cases (staff numbers, perceived care quality), relatives also either withdraw strongly from the care and relinquish responsibility (*Cases A, B*) or feel even more pressure to take on a stronger advocacy role so that adequate care for their relatives can take place at the care unit (*Cases C, D*).

Finally, the problems identified in dealing with stress experienced by other residents differ across the cases. While in *Cases A* and* B,* efforts to raise awareness among other residents are experienced as challenging, in *Cases C* and *D,* "*disruptive*" residents are isolated from group activities, although this is also seen as problematic and dissatisfactory.

#### 5) Using and sharing knowledge

To increase knowledge about dementia that helps to deal with dementia-specific requirements and to provide person-centered dementia care, the following care practices were described by relatives and professionals *in all cases*:

- Expanding staff knowledge (providing dementia-specific training courses; enabling further specializations in dementia-specific topics)
- Learning from other colleagues (internal tips and feedback; contacting medical specialists)
- Seminars for relatives (having exchange platforms; learning new measures)

Nevertheless, using and sharing knowledge about dementia is seen as an existing problem by professionals and relatives *across all cases*. First, there is a huge lack of basic training for nursing assistants and non-qualified staff (supporting every-day activities and cleaning) *in all cases*, who have not acquired sufficient basic knowledge about dementia and techniques for interacting with residents, which makes it difficult for them to understand the disease. This is problematic if the basis of the residents' behaviors cannot be understood or if the appropriateness of the staff’s own behavior cannot be reflected upon (*Cases A, B, D*). Second, existing training for nursing staff is often not offered early enough for new colleagues, is too complicated and does not consider the individuality of residents living with dementia (*Cases B, C*). This leads to uncertainty and excessive demands in care situations as well as staff on site being unable to advise other staff, relatives or residents on how to deal with residents living with dementia and to understand their behavior. Non-person-centered interactions with residents living with dementia can be a consequence of both issues.

*„I found that a bit sad and I was also a bit angry about it because, in my opinion, it's simply wrong to treat people living with dementia like that. But it wasn't malicious on the part of my colleagues. It was simply a lack of knowledge, maybe “* (Case D, professional 17).

##### Differences between the cases

Comparing the cases, the knowledge components are perceived to be better addressed in *Cases A* and *B* than in *Cases C* and *D*. In *Cases C* and *D*, the current level of knowledge is considered lower and is generally described as problematic within the team, as nursing staff rarely receives any further dementia-specific training, and there is no specialized nurse working in the care unit, who can easily be consulted.

## Discussion

Although a variety of care practices are described to deal with dementia-specific requirements in our study, dementia care is still perceived as highly problematic and not person-centered. The existing problems identified in dementia care relate to 1) *handling neuropsychiatric symptoms*, 2) *dealing with communication difficulties*, 3) *providing person-centered interaction and communication*, 4) *dealing with stress caused by experiencing dementia-specific symptoms* and 5) *using and sharing knowledge*. Even though these problems were found in all cases, we were able to identify differences in the extent and perception of these problems across the analyzed cases. The illustration of these context-specific and cross-context problems provides valuable information that need to be considered when developing interventions to improve long-term dementia-specific and person-centered care.

### Similarities between the cases

Our results illustrate that caring for residents living with dementia contribute to more complex and demanding care. Correspondingly, in all cases, the care practices reported to meet dementia-specific requirements include more than just basic care or social activities, and can be mostly assigned to the quality measures described in the National Expert Standard "Fostering and sustaining relationships in care for people living with dementia “ [10]. Furthermore, the care practices mentioned implicitly embed aspects of person-centered care [12, 13], even if they were not named as “resident-centered “ or “person-centered” by any of the participants or in the nursing records. This is consistent with the findings of Colomer and de Vries [43], who showed that although nursing assistants in Ireland indirectly described person-centered interventions in their care activities, they did not identify and name them as “person-centered”. In particular, a lack of knowledge and understanding of person-centeredness was cited as a reason for this [43]. These aspects also played a central role in our cases.

The identified problems are in all cases based on the fact that known care practices are not considered helpful or cannot be implemented to a sufficient extent. Across the cases, this is primarily due to the underlying general context conditions in German nursing homes. The relevance of these conditions is in line with the theoretical model of person-centered care – although not dementia-specific – from McCormack and McCance [44], which explains relevant prerequisites and environmental factors to implement person-centered processes. In addition to the existing lack of staff and time, our findings showed that the problems in *using and sharing knowledge* as well as *dealing with stress caused by experiencing dementia-specific symptoms* can be understood as problematic aspects of the prerequisites and the working environment. Accordingly, the problems in these subject areas can be assigned to the categories of professional competence, appropriate skill mixes, effective working relationships, joint decision-making and supportive organizational structures [44].

Similarly, international studies confirm the relevance of these general conditions in providing high-quality care for people living with dementia. They argue that problems are mainly based on the need for dementia-specific expertise [22, 45–47], supportive leadership and a supporting working environment [45–47], as well as highly qualified nurses as key persons in person-centered dementia care [45, 46]. Owing to the current staff situation in Germany and initiated changes in staff levels (increasing number of nursing assistants) [3, 4], it is expected that the quota of nurses and nursing specialists will further decrease. Therefore, these new skill mixes and the higher quota of nursing assistants need to be addressed through dementia-specific training, the implementation of highly qualified nurses, clear role allocations and intra- and interprofessional exchange formats to positively influence residents’ care quality as well as nurses’ job satisfaction and self-efficacy [5, 7, 45].

Furthermore, we identified problems in *handling neuropsychiatric symptoms, dealing with communication difficulties and providing person-centered interaction and communication* that relate to the internal level of the implementation of person-centered processes [44] in all cases. These problems indicate that the person living with dementia is not considered holistically, and that resident-specific wishes as well as values are not fully taking into account either in decision-making processes or in everyday care. The identified problems are consistent with the results of international studies regarding the perceived excessive demands in dealing with neuropsychiatric symptoms, the strong focus on group harmony rather than individualized person-centered care, the lack of understanding and interpretation of behaviors and emotions, and partly derogatory and disrespectful interactions and communication [21, 22, 48, 49]. In contrast to other studies [22], rigid care routines, fixation measures and unsuccessful interactions were clearly associated with the residents' behavior by the participants in our cases and the commitment—to provide needs-oriented care for the residents—was basically seen as given. Instead, a lack of effective care practices as well as support structures in the transfer and implementation of the well-known dementia-specific knowledge in everyday life was highlighted. This illustrates that not only formal qualifications, further training and "*a palette of strategies to meet different situations* “ [46] are necessary to improve care. In contrast, competencies that promote a dementia-sensitive approach and understanding [50–53] and allow for the intuitive and situational use of knowledge to respond directly to the fluid reactions of residents living with dementia [46, 51, 54] and to timely share their observations with the professional team [49] are needed. Thus, teaching models such as hands-on training, coaching, feedback for observed interactions, group discussions and role-playing for better communication and interactions [53–55] and a holistic view of the person [46, 56, 57] should be explicitly considered in new care models. Besides, successful combinations of theoretical knowledge and practical exercises still need to be investigated in research [58].

All care units included in this study were guided by the National Expert Standard and had either written dementia-specific care concepts, which referred to the person-centered approaches of Kitwood (*Cases A, B*) and validation according to Naomi Feil or Nicole Richard (*Cases B, D*), or DCM (*Cases B, C*) as a measure implemented on the institutional level. However, neither these guidelines nor the DCM were mentioned in any of the interviews or documents. Instead, professionals continued to rate the involvement of residents living with dementia in decision-making processes as limited or impossible, and all participants (residents, relatives, and professionals) expressed a strong desire for more individual and needs-oriented care activities in all cases. This is in line with the results of a German study by Nygaard and colleagues [59], who interviewed residents living with dementia and found that tailored, needs-oriented care appears to be necessary.

Fazio and colleagues [47], Kitwood and Brooker [13] as well as Doyle and Rubinstein [60], among others, have also discussed the fact that person-centered care is a concept of action that has thus far been internalized insufficiently and that its implementation is not just a question of implementation strategies but also requires comprehensive cultural change. This was also confirmed in the pilot implementation of the National Expert Standard [61]. Our findings add that despite the underlying guidelines, the understanding of what is considered dementia-specific care in practice often differs from theoretical approaches of person-centered care. Accordingly, some care units continue to have a stronger focus on how to manage behaviors or deal with the outcomes rather than asking “*What is this person expressing, what is causing this reaction, and how can we respond to reduce their distress?*” [47]. Therefore, it seems to be important not only to implement person-centered concepts in practice in a sustainable way through cultural change but also to continuously evaluate and reflect how the concepts are understood and how great the interpretation gap between the theoretical concept and practice is. Regular sessions with trained experts that are supported by the management [46, 53, 62] to reflect on one’s own attitude and the implementation of the concept in everyday practice as well as case conferences that emphasize the lifeworld of both the resident and the staff [63] could contribute to a better understanding of person-centered care. Including these aspects in new care models would further support the creation of an appropriate person-centered culture and organizational mindset as part of collaborative practice development.

### Differences between the cases

Comparing our cases, more problems in dementia-specific care were described in *Cases C* and* D* than in *Cases A* and *B*. These differences may be explained not only by the different general conditions of the care units and nursing homes (e.g., number of beds, staff ratio, availability of a dementia-specific care concept) but also by the respective approach to the topic of dementia, the underlying attitudes of the professionals and the understanding of person-centered care. *Case B* is quantitatively better staffed than the other care units due to an additional financial regulation for a dementia-specific care unit. Additionally, the nursing staff in *Case B* is better trained and has access to a greater number of underlying concepts and dementia-specific activities. *Case A* also has a specialized nurse in geriatric-psychiatry at the care unit level, who provides expertise in dementia care, as well as a person-centered concept that prioritizes the needs of the residents. This is not the case in *Cases C* and *D*, even though *Case C* also has slightly more staff than usual due to an additional agreement for a house community concept. International studies confirm that context conditions in nursing homes such as the education of staff [21, 22, 45], the physical environment and atmosphere [64], the amount of activities [64] and the scope of person-centered approaches guiding the care process [47, 63, 64] can have an impact on the quality of dementia care. Higher qualified staff and a person-centered approach that is in line with theoretical concepts could be identified only in *Case B* – the dementia-specific care unit. One qualitative study has reported the benefits of dementia-specific care units in terms of relationship work [65]. However, a systematic review has not been able to show clear benefits of dementia-specific care units [66]. One reason for this could be that, despite a person-centered care culture and established measures, the same general problems in providing care exist. Accordingly, due to the clientele (mostly residents with severe dementia) and the more frequent occurrence of several crises at the same time as well as the more pronounced communication deficits of the residents living with severe dementia, the included dementia-specific care unit (*Case B*) also reached its limits, so that well-known measures were not sufficient to cope with these situations. Furthermore, a study by Stranz and Sörensdotter [67] showed how differently person-centered approaches, which are based on theoretical concepts, can and should be lived in different contexts according to the existing problems. Thus, the problems identified in our case study provide deeper insight into cross-context topics that should be included as core elements as well as context-specific subtopics that should be operationalized as peripheral elements in new interventions [68]. For the development of a new dementia-specific, nurse-led care model, it seems, therefore, elementary to consider the necessary flexibility that the care model should have so that existing strengths can be taken into account and relevant context-specific problems can be addressed.

### Strengths and limitations

By using a holistic multiple case study design, we were able to provide in-depth insights into the current situation by describing existing care practices in dementia care and related problems. Our results show how current care can be improved to achieve better outcomes for people living with dementia and the professionals caring for them. Through comparison of the cases, we were further able to present differences between dementia-specific care at the organizational level that have not been investigated before. As our participants were informed and recruited by the nursing home managers, these managers may have been more likely to contact satisfied relatives and residents and/or positive-minded professionals. On the other hand, it is also possible that more critical relatives and residents were more likely to respond to the research team. Both could have influenced the results. The professional sample was further based on a high proportion of nursing staff, as this group was particularly interested in participating. This fact should be reflected in the interpretation of the results, even though it allowed us to focus more on the perspectives of nurses, whose insight into dementia care is particularly important for the development of a dementia-specific nurse-led care model. Furthermore, we were not able to include all professional groups in every case because of a lack of willingness to participate. Finally, we did not include observations as part of our data collection, as this would have been very time-consuming. However, observational data could broaden our findings and should be considered in future studies.

## Conclusion

Despite existing recommendations and care practices used for applying person-centered dementia care in the included nursing homes, current dementia care is perceived as problematic and not person-centered. This highlights that there is still a gap between theoretical knowledge and transformation of that knowledge into practice. Therefore, future research and practice development should give greater consideration to intervention aspects that help staff to use theoretical knowledge as well as existing and well-known care practices during acute crises and to scrutinize the current understanding of dementia-specific care to promote a person-centered attitude and care culture. Moreover, the identified cross-case differences show that the design of new care models should allow for more flexibility so that these models can be adapted to given contexts and their success can be evaluated realistically in comparison to usual care, which is anything but homogeneous.

## Supplementary Information

Additional File 1: Reporting guideline for organizational case studies.

Additional File 2: Interview guidelines.

Additional File 3: Context questionnaire (German version).

## Data Availability

The datasets used and/or analyzed during the current study are available from the corresponding author on reasonable request.

## Abbreviations

DemCare-Q: Dementia Care Questionnaire
DCM: Dementia Care Mapping
DSS: Dementia Screening Scale
