# Supplementary material for: Provision of dementia-specific care in nursing homes in North Rhine-Westphalia (Germany) – analysis of person-centered practices and related problems within a holistic multiple case study
Source: BMC Nurs. 2025 Feb 1;24:116. doi: 10.1186/s12912-025-02726-5 (PMC11786518; doi:10.1186/s12912-025-02726-5)
Supplement: Supplementary file 1 — Additional File 1: Reporting guideline for organizational case studies. [file 12912_2025_2726_MOESM1_ESM.pdf]

Additional file 1

Consensus standards for the reporting of organizational case studies

Reporting item		Page number on which item was reported	Page number of justification for not reporting
Describing the design			
1	Define the research as a case study	Titel; Abstract; 3	
2	State the broad aims of the study	3	
3	State the research question(s)/hypotheses	3	
4	Identify the specific case(s) and justify the selection	3-4	
Describing the data collection			
5	Describe how data were collected	4-5	
6	Describe the sources of evidence used	4-5	
7	Describe any ethical considerations and obtainment of relevant approvals, access and permissions	5	
Describing the data analysis			
8	Describe the analysis methods	5	
Interpreting the results			
9	Describe any inherent shortcomings in the design and analysis and how these might have influenced the findings	15	
10	Consider the appropriateness of methods used for the question and subject matter and why it was that qualitative methods were appropriate	15	
11	Discuss the data analysis	15	
12	Ensure that the assertions are sound, neither over- nor under-interpreting the data	13-15	
13	State any caveats about the study	15	

Rodgers, M., Thomas, S., Harden, M., Parker, G., Street, A., & Eastwood, A. (2016). Developing a methodological framework for organisational case studies: a rapid review and consensus development process. *Health Services and Delivery Research, 4*(1), 1-170. https://doi.org/10.3310/hsdr04010
