# Supplementary material for: Provision of dementia-specific care in nursing homes in North Rhine-Westphalia (Germany) – analysis of person-centered practices and related problems within a holistic multiple case study
Source: BMC Nurs. 2025 Feb 1;24:116. doi: 10.1186/s12912-025-02726-5 (PMC11786518; doi:10.1186/s12912-025-02726-5)
Supplement: Supplementary file 2 — Additional File 2: Interview guidelines. [file 12912_2025_2726_MOESM2_ESM.pdf]

Interview guide – professionals

Topic	Questions
**Interview aim**	**Brief introduction:** We would like to develop an idea of what a care model in residential long-term care could look like - to this end, we would like to reflect with you how care of residents living with dementia is currently provided here in the care unit.
**Introduction**	**Please think about one or two residents with dementia of the care unit.**<br>**In your opinion, what special requirements apply to the care of residents with dementia compared to the care of residents without dementia?**<br>Are there differences in care planning or care design?<br>What is different?
**Involvement**	**To what extent is the resident with dementia involved in care?**<br>Are the personal preferences or wishes of the resident with dementia recorded and if so, how?<br>To what extent is the resident involved in decision-making / planning?<br>What are the reasons for not involving residents with dementia in decision-making / planning?<br>**To what extent are relatives involved in care?**<br>To what extent are relatives involved in decision-making / planning?<br>What are the reasons for not involving relatives in decision-making / planning?
**Interaction / communication / relationship**	**How do you interact and communicate with the resident with dementia?**<br>What topics do you talk about with the resident?<br>How would you describe your relationship with the resident?<br>**How do you interact and communicate with relatives?**<br>What topics do you talk about with the relatives of the resident?<br>How would you describe your relationship with the relatives of the resident?
**Problems in dementia-specific care**	**Were there situations in which the resident's needs or wishes were not seen/not recorded?**<br>What were the situations? What were the problems?<br>What would have helped you?<br>What were the results of the situations? (Outcomes)<br>**Were there situations in which you did not understand the resident/in which communication with the resident was not possible?**<br>What were the situations? What were the problems?<br>What would have helped you?<br>What were the results of the situations? (Outcomes)
**Measures for dementia-specific care**	**Are there specific measures already being taken to promote the care of the resident with dementia?**<br>What do you understand by the specific measures? How do you structure them?<br>How do you feel about these measures? (helpful - problematic)<br>What need for support do you see?<br>**Are there specific measures already being taken to promote interaction with relatives?**<br>How do you feel about these measures? (helpful - problematic)<br>What support do you need?
**Problems and strengths in general**	**What strengths and problems do you currently perceive in care overall? Can you tell me about them?**<br>What is currently working very well? What should remain as it is?<br>What is working less well? Where are problems?

	Are there specific "weaknesses" in the care of people with dementia? Tell me about them.
**Vision of care**	**If care in the care unit would meet your expectations, what would good care look like?**<br>What does "good" care mean to you?
Changes needed	**In your opinion, to what extent does the current care fulfill this expectation? What changes do you think are necessary to achieve this?**<br>What would be the most important goal of change?<br>What would be additionally desirable but less important?
**Closing**	**Are there any other things you'd like to mention on the topic that we haven't addressed so far?**

Interview guide – relatives

Topic	Questions
**Introduction**	**I would like to know more about how your relative is receiving care and being cared for here in this care unit. I would like you to tell me about the care from your point of view.**<br>What does a typical day look like?<br>What do you consider to be part of everyday care?
**Problems and strengths in general**	**Please tell me three aspects of care in the care unit that you think work really well.**<br>Are there other things that you are currently very satisfied with?<br>Which other things should not change?<br>**Which three aspects are working less well?**<br>What do you think is the biggest problem?<br>What are other problems?<br>Where do you see problems in dementia care?
**Involvement**	**When you think about the individual needs, wishes and preferences of your relative. Would you say that these are taken into account in the care provided?**<br>If so: How and when does this happen?<br>What contributes to your feeling that your relative's wishes are perceived / not perceived by the staff?<br>**Do you think that your relative is also involved in care planning or decision-making? Can you explain this to me?**<br>**To what extent are you yourself involved in the care and its planning?**<br>Who will talk to you about your relative's problems or acute complaints?<br>Who informs you about decisions made? Who discusses these with you?
**Interaction / communication**	**Sometimes there are also problems in communicating with residents with dementia. Have there ever been situations in which you felt that your relative's needs or wishes were not heard or understood?**<br>What kind of situations were that? Can you describe them to me?<br>What would you have wished for?<br>**Additionally, the exchange of information between relatives and staff can also be problematic. Were there any situations in which you as a relative did not feel heard or understood?**<br>What kind of situations were these? Can you give me an example?<br>What would you have wished for?

**Relationship**	**If you think about the different professional groups involved in the care here (nurses, physicians, therapists). How would you describe your relationship with the different people here?**<br>Which member of staff do you have the best relationship with?<br>Who do you trust the most?<br>How do you experience the relationship between the various staff and your relative?<br>What would you wish for?<br>**Have there ever been situations in which you feel that your relative did not feel accepted as a person?**<br>What were these situations?<br>What would you have wished for your relative?
**Vision of care**	**Finally, if you imagine that the care here in the care unit would meet your expectations. What would good care look like?**<br>What does "good" mean to you in this case?<br>To what extent does the current care fulfill this in your opinion?
Changes needed	**What changes in the design of care would you like to see?**<br>What would be the most important goal of change?<br>What would be additionally desirable but less important?<br>What should not be changed?
**Closing**	**Are there any other things you'd like to mention on the topic that we haven't addressed so far?**

Interview guide – residents with dementia

**Topic**	**Questions**
**Introduction**	**I am interested in how you experience your care here (in the care unit). When you think about your daily care, what is it like here?**<br>How is a day like here?<br>What is part of your everyday life?<br>What do you do in the morning / at lunchtime / in the evening?
**Problems and strengths in general**	**What do you like best about your care here?**<br>**What do you not like here at all?**<br>What works well here?<br>What should stay that way?<br>What are the problems?<br>What is the biggest problem here?
**Involvement**	**Is there anything that should be considered in your care? Something that is particularly important to you?**<br>What is that?<br>Have you ever told this to the nurses or physicians?<br>I heard that you like ____________________. Is that true?<br>**Are these wishes (_______________) currently being taken into account?**<br>Yes: How are they taken into account? When does this happen?<br>No: What should be paid more attention to?
**Interaction / communication**	**What if no one understands what you want? Does that happens?**<br>What kind of situations are these?<br>What do you do then? How do you deal with them?<br>What would you wish for in such situations?

	**Are there also situations in which the nursing staff or physicians grumble at you?**<br>What kind of situations are these?<br>What do you do then? How do you deal with it?<br>What would you wish for in such situations?
**Relationship**	**Who do you talk to most often?**<br>Who do you talk to when you are not feeling well?<br>Who helps you?<br>Who do you talk to about personal issues?<br>Is there anyone you don't like talking to? Why?<br>**Do you also talk to the other residents?**
**Vision of care**	**What would good care look like for you?**<br>What does "good" mean to you?<br>Is the care here currently "good"?
Changes needed	**What changes would you like to see?**<br>What would be the most important goal?<br>What else would be important?<br>What should remain the same?<br>**What would you like to see from the nurses / physicians / therapists?**
**Closing**	**Are there any other things you would like to mention?**
