# Supplementary material for: Provision of dementia-specific care in nursing homes in North Rhine-Westphalia (Germany) – analysis of person-centered practices and related problems within a holistic multiple case study
Source: BMC Nurs. 2025 Feb 1;24:116. doi: 10.1186/s12912-025-02726-5 (PMC11786518; doi:10.1186/s12912-025-02726-5)
Supplement: Supplementary file 3 — Additional File 3: Context questionnaire (German version). [file 12912_2025_2726_MOESM3_ESM.pdf]

FRAGEBOGENERHEBUNG KONTEXT

Informationen zur Pflegeeinrichtung und Wohneinheit

Datum der Erhebung: __________(TT.MM.JJJJ)

Code Einrichtung: E ___

Code Wohnbereich: E ___[W __]

STRUKTURFRAGEBOGEN PFLEGEEINRICHTUNG

1. **Zu welcher Trägerform gehört Ihre Einrichtung?**
   - ☐ Frei-gemeinnützig
   - ☐ Öffentlich/Kommunal
   - ☐ Privat

2. **Wie viele Einwohner*innen hat der Ort, in dem ihre Einrichtung liegt? (gemeint ist die Stadt, nicht der Stadtteil bzw. die Kommune nicht der Ortsteil)**
   - ☐ Weniger als 20.000
   - ☐ 20.000 – 100.000
   - ☐ Über 100.000
   - ☐ Über 1 Million

3. **Bitte machen Sie Angaben zur Angebotsstruktur Ihrer Einrichtung**

   Anzahl Wohnbereiche: ________

   Anzahl vollstationärer Plätze: _________

   Anzahl Plätze in der Kurzzeitpflege (definiert danach, wie sie abgerechnet werden)

   Solitär: _________ Eingestreut: __________

4. **Wann wurde die Einrichtung gebaut (bezieht sich auf den Bau des erstmalig genutzten Gebäudeteils)**

   Jahr: _________

5. **Wie viele Vollzeitstellen für Pflegefachkräfte stehen für diese Einrichtung zur Verfügung?**

   Anzahl (SOLL): ______Vollzeitstellen

6. **Wie viele Stellen sind davon aktuell besetzt?**

   Anzahl (IST):________Vollzeitstellen

7. **Gibt es in der Einrichtung ein theoretisch fundiertes, schriftlich niedergelegtes demenz-spezifisches Betreuungskonzept?**
   - ☐ Nein
   - ☐ Ja
   - ☐ Weiß ich nicht

a. **Welche der folgenden Ansätze sind in dem Betreuungskonzept benannt? (Mehrfachnennung möglich)**

☐ Personenzentrierter Ansatz nach Tom Kitwood

☐ Psychobiografischer Ansatz nach Erwin Böhm

☐ Validation nach Cora an der Koij/Naomi Feil/Nicole Richard

☐ Milieutherapeutischer Ansatz

☐ Normalisierungsprinzip/Alltagsnormalität

☐ Es ist kein Ansatz explizit benannt

☐ Sonstiges: _______________

b. **Welche der folgenden konkreten Maßnahmen sind Bestandteil des Demenzkonzeptes? (Mehrfachnennung möglich)**

☐ Durchführung von Fallbesprechungen

☐ Verhaltens- und kognitionsbezogene Assessments

☐ Validation

☐ Erinnerungspflege/Biografiearbeit

☐ Multisensorische Stimulation (z.B. Basale Stimulation; Snoezeln)

☐ Bewegungsförderung

☐ Sonstiges: _____________________

STRUKTURFRAGEBOGEN WOHNEINHEIT

8. **Wie kennzeichnet sich ein Wohnbereich in Ihrer Pflegeeinrichtung?**

____

____

____

____

Teil A: Architektur

9. **Wurde der Wohnbereich speziell für Menschen mit Demenz gebaut?**

☐ Nein

☐ Ja

☐ Weiß ich nicht

10. **Ist der Wohnbereich baulich von anderen Wohnbereichen abgetrennt?**

☐ Nein

☐ Ja

☐ Weiß ich nicht

11. Ist der Wohnbereich ein Wohnbereich mit Aus- und Zugangskontrollen (beschützter Bereich)?

☐ Nein

☐ Ja

☐ Weiß ich nicht

12. Liegt der Wohnbereich auf mehreren Etagen?

☐ Nein

☐ Ja

☐ Weiß ich nicht

13. Ist der Wohnbereich in verschiedene Wohngruppen unterteilt?

☐ Nein

☐ Ja, in_________ Wohngruppen

☐ Weiß ich nicht

14. Verfügt der Wohnbereich über einen eigenen unmittelbar vom Wohnbereich aus zugänglichen Außenbereich (z. B. Garten, Terrasse, Dachterrasse, Balkon), den Bewohner*innen aller Wohngruppen nutzen können?

☐ Nein

☐ Ja

☐ Weiß ich nicht

15. Hat der Wohnbereich einen eigenen Pflegestützpunkt?

☐ Nein

☐ Ja

☐ Weiß ich nicht

16. Wie viele Bewohnerzimmer hat der Wohnbereich?

Anzahl:_________ Zimmer

17. Wie viele Bewohnerzimmer sind davon Einzelzimmer?

Anzahl:_________ Zimmer

18. Wann wurden die letzten umfassenden Anbau-, Umbau- oder Renovierungsarbeiten im Wohnbereich vorgenommen?

☐ In den letzten 1-2 Jahren

☐ Vor 3-5 Jahren

☐ Vor 6-10 Jahren

☐ Vor über 10 Jahren

☐ Gar nicht (z. B. weil Neubau)

☐ Unbekannt

a. **Welche Maßnahmen umfassten die Anbau-, Umbau- oder Renovierungsarbeiten?**

____

____

____

Teil B: Finanzierung

19. **Ist für den Wohnbereich ein pflegefachlicher Schwerpunkt vertraglich mit dem Kostenträger vereinbart?**

☐ Nein

☐ Ja

☐ Weiß ich nicht

a. **Wenn ja, welcher pflegefachliche Schwerpunkt?**

☐ Demenz

☐ Gerontopsychiatrie

☐ Beschützter Bereich (mit Unterbringungsbeschluss)

☐ Anderer: ____________________

b. **Sind Aufnahmekriterien mit dem Kostenträger vereinbart?**

☐ Nein

☐ Ja, und zwar folgende: __________________

☐ Weiß ich nicht

20. **Wurde für den Wohnbereich eine besondere Vergütungsvereinbarung mit den Kostenträgern abgeschlossen?**

☐ Nein

☐ Ja

☐ Weiß ich nicht

a. **Liegen die Kosten für den/die Bewohner*in für diesen Wohnbereich über denen der anderen Wohnbereiche?**

☐ Nein

☐ Ja

☐ Weiß ich nicht

b. **Wurde mit dem Kostenträger für diesen Wohnbereich ein höherer Personalschlüssel für Pflegefachkräfte vereinbart als für die anderen Wohnbereiche der Einrichtung?**

☐ Nein

☐ Ja

☐ Weiß ich nicht

Teil C: Personal

21. Bitte geben Sie an, welche und wie viele Personen in den folgenden Qualifikationsgruppen an der Versorgung der Bewohner*innen des Wohnbereichs regulär beteiligt sind.

- ☐ Pflegefachkräfte (3-jährige Ausbildung) Anzahl: ____ Vollzeitäquivalent: ____
- ☐ Pflegefachkräfte mit Weiterbildung Anzahl: ____ Vollzeitäquivalent: ____
  und zwar: ___________________
- ☐ Pflegehilfskräfte (mind. 1-jährige Ausbildung) Anzahl: ____ Vollzeitäquivalent: ____
- ☐ Pflege: ungelernte Mitarbeiter*innen Anzahl: ____ Vollzeitäquivalent: ____
- ☐ Pflege: Auszubildende Anzahl: ____ Vollzeitäquivalent: ____
- ☐ Hauswirtschaftskräfte Anzahl: ____ Vollzeitäquivalent: ____
- ☐ Mitarbeiter*innen des Sozialen Dienstes Anzahl: ____ Vollzeitäquivalent: ____
- ☐ Betreuungskräfte Anzahl: ____ Vollzeitäquivalent: ____
- ☐ Therapeutisches Personal Anzahl: ____
  und zwar: ___________________
- ☐ Ehrenamtliche Anzahl: ____
- ☐ Hausärzt*innen Anzahl: ____
- ☐ Fachärzt*innen Anzahl: ____
  und zwar: ___________________
- ☐ Sonstige: _________________________ Anzahl: ____

22. Sind die Pflegefachkräfte dem Wohnbereich fest zugeordnet?

- ☐ Nein
- ☐ Ja
- ☐ Weiß ich nicht

23. Ist im Nachtdienst kontinuierliche eine Pflegefachkraft anwesend, die nur für diesen Wohnbereich zuständig ist?

- ☐ Nein
- ☐ Ja
- ☐ Weiß ich nicht

24. Ist außerhalb des Nachtdienstes kontinuierlich eine Pflegefachkraft anwesend?

- ☐ Nein
- ☐ Ja
- ☐ Weiß ich nicht

25. Hat die pflegefachliche Leitung des Wohnbereichs eine Weiterbildung zur Fachkraft für Gerontopsychiatrie absolviert?

- ☐ Nein
- ☐ Ja

☐ Weiß ich nicht

26. Ist die pflegefachliche Leitung des Wohnbereichs in Vollzeit beschäftigt?

☐ Nein

☐ Ja

☐ Weiß ich nicht

27. Ist die pflegefachliche Leitung des Wohnbereichs für mehr als einen Wohnbereich zuständig?

☐ Nein

☐ Ja

☐ Weiß ich nicht

Teil D: Bewohner*innen

28. Bitte geben Sie an, wie viele Bewohner*innen insgesamt in dem Wohnbereich leben.

Anzahl:__________ Bewohner*innen

29. Bitte geben Sie an, wie viele Bewohner*innen davon Kurzzeitpflegegäste sind.

Anzahl:__________ Kurzzeitpflegegäste

30. Bitte geben Sie an, wie viele Bewohner*innen den unterschiedlichen Pflegegraden zugeordnet sind (nur vollstationär).

Pflegegrad 0: _________Bewohner*innen / Prozentualer Anteil: ___ %

Pflegegrad 1: _________Bewohner*innen / Prozentualer Anteil: ___ %

Pflegegrad 2: _________Bewohner*innen / Prozentualer Anteil: ___ %

Pflegegrad 3: _________Bewohner*innen / Prozentualer Anteil: ___ %

Pflegegrad 4: _________Bewohner*innen / Prozentualer Anteil: ___ %

Pflegegrad 5: _________Bewohner*innen / Prozentualer Anteil: ___ %

31. Bitte schätzen Sie, wie viele Bewohner*innen des Wohnbereichs eine ärztliche Demenzdiagnose haben.

Anzahl:_________ Bewohner*innen ODER Prozentualer Anteil: ___ %

32. Bitte schätzen Sie, wie viele Bewohner*innen nicht mehr aus dem Bett mobilisiert werden können.

Anzahl:_________ Bewohner*innen ODER Prozentualer Anteil: ___ %

33. Bitte geben sie an, für wie viele Bewohner*innen ein richterlicher Unterbringungsbeschluss für die Anwendung von freiheitseinschränkenden Maßnahmen vorliegt.

Anzahl:_________ Bewohner*innen

34. Bitte geben Sie an, für wie viele Bewohner*innen ein richterlicher Beschluss für die Anwendung von körpernahen freiheitseinschränkenden Maßnahmen vorliegt.

Anzahl:_________ Bewohner*innen

Teil E: Mahlzeiten

35. Haben die Bewohner*innen die Möglichkeit, das Mittagessen gemeinsam im Wohnbereich einzunehmen?

☐ Nein

☐ Ja

☐ Weiß ich nicht

36. Wird das Mittagessen grundsätzlich im Wohnbereich zubereitet (d.h. Kochen, Erhitzen, Vorbereitung von Mahlzeiten)?

☐ Nein

☐ Ja

☐ Weiß ich nicht

37. Wie wird das Mittagessen überwiegend serviert?

☐ Mahlzeiten werden auf einem Tablett angeliefert und dem/der Bewohner*in ausgeteilt

☐ Mahlzeiten werden in Schüsseln/Töpfen angeliefert und auf Tellern angerichtet

☐ Tisch wird gedeckt wie zuhause: Mahlzeiten stehen zur Selbstbedienung auf dem Tisch

☐ Anderes:___________________

Teil F: Versorgung und Betreuung

38. Führen Sie in dem Wohnbereich Fallbesprechungen durch?

☐ Nein

☐ Ja

☐ Weiß ich nicht

a. Was kennzeichnet durchgeführte Fallbesprechungen? (Mehrfachnennung möglich)

☐ Fallbesprechungen folgen einem festgelegten Ablauf

☐ Mitglieder*innen verschiedener Berufsgruppen werden hinzugezogen

☐ Bei Bedarf wird ein*e externe*r Moderator*in hinzugezogen

☐ Bewohner*innen bzw. Angehörige werden eingeladen

☐ Ergebnisse der Fallbesprechungen werden protokolliert

☐ Ergebnisse der Fallbesprechungen werden evaluiert

☐ Die teilnehmenden Mitarbeiter*innen haben während der Fallbesprechung keine anderen Aufgaben zu erledigen

b. Wer wird in der Regel zu Fallbesprechungen eingeladen? (Mehrfachnennung möglich)

☐ Bewohner*innen

☐ Angehörige

☐ Gesetzliche Betreuer*innen

☐ Heimleitung/Pflegedienstleitung

☐ Mitglieder*innen des Pflegeteams des Wohnbereichs

☐ Weiteres Betreuungspersonal (z.B. Mitarbeiter*innen des Sozialen Dienstes)

☐ Hausärzt*innen

☐ Fachärzt*innen, und zwar:

☐ Therapeutisches Personal, und zwar: _______________

☐ Mitarbeiter*innen aus Hauswirtschaft/Küche

☐ Externe*r Moderator*in

☐ Sonstiges: _______________

c. **Wann werden Fallbesprechungen in der Regel durchgeführt?**

☐ Regelmäßig in festen zeitlichen Abständen

☐ Anlassorientiert und von daher unregelmäßig

☐ Beides

d. **Wo werden Fallbesprechungen in der Regel durchgeführt?**

☐ In einem Besprechungsraum

☐ Im Dienstzimmer

☐ Im Personalaufenthaltsraum

e. **Wie häufig kommt es vor, dass Teilnehmer*innen während der Fallbesprechung diese verlassen möchten, um Aufgaben im Wohnbereich zu erledigen?**

☐ Nie

☐ Selten

☐ Manchmal

☐ Immer

39. Wird im Wohnbereich regelmäßig (mindestens einmal im Jahr) Dementia Care Mapping von einer Person durchgeführt, die nicht in dem Wohnbereich arbeitet?

☐ Nein

☐ Ja

☐ Weiß ich nicht

40. Werden in dem Wohnbereich standardisierte Instrumente zur Einschätzung von Schmerzen eingesetzt?

☐ Nein

☐ Ja, bei manchen Bewohner*innen

☐ Ja, bei allen Bewohner*innen

a. **Mit welchen Instrumenten wird in der Wohneinheit die Einschätzung von Schmerzen bei den Bewohner*innen vorgenommen? (Mehrfachnennung möglich)**

☐ Nummerische Rangskala (NRS)

☐ Visuelle Analogskala (VAS)

☐ Verbale Rangskala (VRS)

☐ Smiley Scale

☐ Face Pain scale

☐ Beurteilung von Schmerzen bei Demenz (BESD)

☐ Beobachtungsinstrument für das Schmerzassessment bei alten Menschen mit Demenz (BISAD)

☐ Echelle comportementale de la douleur pour personnes ágées non communicate (ECPA)

☐ Zürich Observation Pain Assessment (ZOPA)

☐ Behavioural Pain Assessment in the elderly (Dolopus 2)

☐ Wir nutzen ein selbst entwickeltes Instrument

☐ Sonstiges: _______________

41. **Werden in dem Wohnbereich standardisierte Instrumente zur Einschätzung des Verhaltens eingesetzt?**

☐ Nein

☐ Ja, bei manchen Bewohner*innen

☐ Ja, bei allen Bewohner*innen

a. **Mit welchen Instrumenten wird in der Wohneinheit die Einschätzung des Verhaltens bei den Bewohner*innen vorgenommen? (Mehrfachnennung möglich)**

☐ Resident Assessment Instrument (RAI)

☐ Cohen-Mansfield Assessment-Instrument (CMAI)

☐ Nurses' Observation Scale for Geriatric Patients (NOSGER)

☐ Innovatives Demenzorientiertes Assessmentsystem (IDA)

☐ Neuropsychiatrisches Inventar (NPI)

☐ Wir nutzen ein selbst entwickeltes Instrument

☐ Sonstiges: _______________

42. **Wird bei den Bewohner*innen mit Demenz/Verdacht auf Demenz in dem Wohnbereich eine Einschätzung des Demenzschweregrades durchgeführt?**

☐ Nein

☐ Ja

a. **Durch wen wird diese Einschätzung in der Regel vorgenommen?**

☐ Durch den betreuenden Arzt/die betreuende Ärztin

☐ Durch die Pflegenden in der Einrichtung

☐ Unterschiedlich

☐ Weiß ich nicht

b. Mit welchen Instrumenten wird die Einschätzung der Demenzschwere bei den Bewohner*innen vorgenommen? (Mehrfachnennung möglich)

☐ Mini-Mental-State Examination (MMST)

☐ Global Deterioration Scale (GDS)

☐ Clinical Dementia Rating (CDR)

☐ Alzheimer's Disease Assessment Scale (ADAS)

☐ CERAD-NP-Testbatterie

☐ Uhren-Test

☐ Functional Assessment Staging (FAST)

☐ DEM-TECT

☐ Strukturiertes Interview für die Diagnose einer Demenz (SIDAM)

☐ Wir nutzen ein selbst entwickeltes Instrument

☐ Sonstiges: _______________

43. Werden in dem Wohnbereich standardisierte Instrumente zur Einschätzung der Lebensqualität eingesetzt?

☐ Nein

☐ Ja, bei manchen Bewohner*innen

☐ Ja, bei allen Bewohner*innen

a. Mit welchen Instrumenten wird in der Wohneinheit die Einschätzung der Lebensqualität bei den Bewohner*innen vorgenommen? (Mehrfachnennung möglich)

☐ QUALIDEM

☐ Dementia Quality of Life (DQoL)

☐ DEMQOL

☐ Quality of Life Alzheimer Disease (QOL-AD)

☐ Heidelberger Instrument zur Erfassung der Lebensqualität Demenzkranker (HILDE)

☐ CLIPPER

☐ Dementia Care Mapping (DCM)

☐ Alzheimer Disease Related Quality of Life (ADRQL)

☐ Discomfort Scale-Dementia of Alzheimer Type (DS-DAT)

☐ Quality of life on late Stage dementia (QUALID)

☐ Profil des Wohlbefindens

☐ Wir nutzen ein selbst entwickeltes Instrument

☐ Sonstiges: _______________

44. Werden in dem Wohnbereich standardisierte Instrumente zur Einschätzung von Depressionen der Bewohner*innen eingesetzt?

☐ Nein

☐ Ja, bei manchen Bewohner*innen

☐ Ja, bei allen Bewohner*innen

a. Mit welchen Instrumenten wird in der Wohneinheit die Einschätzung von Depressionen bei den Bewohner*innen vorgenommen? (Mehrfachnennung möglich)

☐ Geriatrische Depressionskala (GDS)

☐ Hamilton Depressionskala

☐ Wir nutzen ein selbst entwickeltes Instrument

☐ Sonstiges: _______________
